# Letter to the Editor about Artificial intelligence predicts multiclass molecular signatures and subtypes directly from breast cancer histology: a multicenter retrospective study

**DOI:** 10.1097/JS9.0000000000003303

**Published:** 2025-08-25

**Authors:** Kun Fang, Suxiao Jiang, Chunhua Mao

**Affiliations:** a Department of Surgery, Yinchuan Maternal and Child Health Hospital, Yinchuan, China; b Department of Surgery, Yinchuan Maternal and Child Health Hospital Affiliated to Ningxia Medical University, Yinchuan, China

*Dear Editor,*

We have read with great interest the recent article published in your journal titled “Artificial intelligence predicts multiclass molecular signatures and subtypes directly from breast cancer histology: a multicenter retrospective study”^[1]^. This study presents an innovative approach using H&E-stained histopathology slides to predict multiple molecular markers and novel prognostic subtypes of breast cancer through deep learning techniques, aiming to reduce the cost and tissue consumption of conventional assays. While the study holds significant clinical promise, we would like to raise several methodological and interpretative concerns for further consideration.

First, although the deep learning-based algorithm (BBMIL) model demonstrated favorable area under curve (AUC) values in the The Cancer Genome Atlas Breast Invasive Carcinoma (TCGA-BRCA) and several external datasets – particularly for classic markers such as estrogen receptor-negative (ER) and progesterone receptor (PR), the performance for human epidermal growth factor receptor 2 (HER2) prediction remains suboptimal, with an AUC of 0.641 in the validation cohort and 0.595 in the Early Breast Cancer Core-Needle Biopsy WSI (BCNB) biopsy dataset. These results fall short compared to current clinical diagnostic standards and other similar studies^[2–4]^. Given the importance of HER2 in guiding targeted therapies in breast cancer, the relatively low accuracy hinders the model’s clinical applicability. While the authors acknowledged the challenges of HER2 prediction in prior studies, they did not provide a detailed discussion of possible causes or potential solutions, such as the limited morphological features associated with HER2 expression, high intratumoral heterogeneity, or annotation inconsistencies.

Second, although automatic segmentation of cancer regions of interest (ROI) significantly enhanced model performance, the BBMIL-NA (non-annotated) version still showed competitive results. This suggests that precise delineation of tumor areas may not be strictly necessary. It would be valuable for the authors to quantify how different ROI annotation strategies influence model generalizability, especially under variable clinical conditions such as sample heterogeneity or differences in scanning quality.

Third, the study included predictions of five immune-related gene signatures and two newly proposed prognostic subtypes (Tumor Immunophenotyping [TIP] and cell death-associated subtype). However, the biological rationale and clinical implications of these subtypes were not thoroughly explored. Mechanistic insight and large-scale clinical validation are currently lacking. Moreover, stratification based on immune infiltration and survival appears to rely primarily on computational inference, which requires further validation using multimodal data and functional experiments to improve model credibility and translational value.

Fourth, while the use of Transformer and attention mechanisms in the BBMIL model design may enhance instance-level relationship learning, the manuscript lacks an in-depth analysis of model interpretability. One of the key barriers to clinical adoption of AI models is the so-called “black box” nature, where the basis for predictions is not clearly visible to clinicians. Future work should consider incorporating visual and quantitative interpretability tools – such as activation maps or feature importance scoring to improve model transparency and facilitate clinical acceptance^[5]^.

Lastly, despite including a relatively large number of samples covering both pre-treatment and biopsy specimens, the datasets were primarily derived from public repositories or single centers. There was limited information on cross-center consistency and quality control, which may affect the generalizability and robustness of the model. Prospective, multi-center studies involving diverse populations and platforms are essential to assess the real-world utility and reliability of such approaches.

In conclusion, this study represents a promising advancement in the prediction of molecular markers and prognostic subtypes of breast cancer using histopathological images. We commend the authors for their efforts. Nevertheless, improvements are warranted in HER2 prediction accuracy, biological validation, model explainability, and clinical applicability across diverse settings. We hope that this work will stimulate further research to refine and validate AI-assisted pathology tools in the context of precision oncology. This study adhered to the TITAN (Transparency In The reporting of Artificial INtelligence) reporting guideline^[6]^.

## Data Availability

Data sharing is not applicable to this article.

## Research registration unique identifying number (UIN)

There are no conflicts of interest.
